# Predictors of colorectal cancer survival using cox regression and random survival forests models based on gene expression data

**DOI:** 10.1371/journal.pone.0261625

**Published:** 2021-12-29

**Authors:** Mohanad Mohammed, Innocent B. Mboya, Henry Mwambi, Murtada K. Elbashir, Bernard Omolo

**Affiliations:** 1 School of Mathematics, Statistics and Computer Science, University of KwaZulu-Natal, Pietermaritzburg, Scottsville, South Africa; 2 Faculty of Mathematical and Computer Sciences, University of Gezira, Wad Madani, Sudan; 3 Department of Epidemiology and Biostatistics, Kilimanjaro Christian Medical University College (KCMUCo), Moshi, Tanzania; 4 College of Computer and Information Sciences, Jouf University, Sakaka, Saudi Arabia; 5 Division of Mathematics & Computer Science, University of South Carolina-Upstate, Spartanburg, United States of America; 6 School of Public Health, Faculty of Health Sciences, University of Witwatersrand, Johannesburg, South Africa; University of Technology Malaysia: Universiti Teknologi Malaysia, MALAYSIA

## Abstract

Understanding and identifying the markers and clinical information that are associated with colorectal cancer (CRC) patient survival is needed for early detection and diagnosis. In this work, we aimed to build a simple model using Cox proportional hazards (PH) and random survival forest (RSF) and find a robust signature for predicting CRC overall survival. We used stepwise regression to develop Cox PH model to analyse 54 common differentially expressed genes from three mutations. RSF is applied using log-rank and log-rank-score based on 5000 survival trees, and therefore, variables important obtained to find the genes that are most influential for CRC survival. We compared the predictive performance of the Cox PH model and RSF for early CRC detection and diagnosis. The results indicate that *SLC9A8*, *IER5*, *ARSJ*, *ANKRD27*, and *PIPOX* genes were significantly associated with the CRC overall survival. In addition, age, sex, and stages are also affecting the CRC overall survival. The RSF model using log-rank is better than log-rank-score, while log-rank-score needed more trees to stabilize. Overall, the imputation of missing values enhanced the model’s predictive performance. In addition, Cox PH predictive performance was better than RSF.

## Introduction

Colorectal cancer (CRC) is the second leading cause of mortality in women and third in men [1]. The American cancer society estimate, about 1 in 23 men and 1 in 25 women develop colorectal cancer in their lifetime [2]. Globally, there were about 19.3 million new cancer cases in 2020 alone, while close to 10 million deaths were recorded due to cancer [3]. CRC represents 9.4% of cancer deaths and 10% of newly diagnosed cancer cases [3]. The incidence and mortality in males are 10.6% and 9.3%, respectively, while the incidence and mortality in females are 9.4% and 9.5%, respectively [3]. Early detection of CRC can reduce mortality due improved chemotherapy regimens and surgical techniques [4–6]. The prognosis and survival of early intervention with CRC patients are linked with tumor staging, where early diagnosis of the tumor is more likely to be curable [7]. The 5-year relative survival rates for patients with localized CRC was 91% in the USA between 2010 and 2016 [8]. However, the 5-year relative survival rates of CRC cases at regional and distant stages are 72% and 14%, respectively [8]. The main characteristics of the CRC are that it has high inter-patient and intra-tumor heterogeneity. Other factors such as environment, lifestyle, and diet can lead to further heterogeneity in the CRC occurrence and progression [9–11]. This heterogeneity leads to variations in response to treatment between individuals. Determining the molecular markers is clinically essential to help detect and precisely predict the prognosis of patients with CRC.

Researchers have developed many methods to determine the prognostic molecular markers to early detect and predict the prognosis of patients with CRC. These methods include univariate and multivariate Cox proportional hazard models, elastic net estimation, and random forests for survival prediction [4, 7, 12–15]. Previous studies such as, Abdul Aziz *et al*. [12] analyzed the CRC death using the Cox proportional hazard model, and they reported a 19 gene signature that could predict the survival of CRC patients with Dukes’ B and C stages. In their work, Abdul Aziz *et al*. used SAM, *limma*, and t-test to identify the most significant genes based on microarray gene expression data. Dai *et al*. [4] conducted a survival analysis using univariate and multivariate Cox models based on three microarray datasets from GEO and one dataset from the TCGA database. They used the DEGs from each of the three microarray datasets, and they identified 105 mutual DEGs based on the intersection of the three DEGs lists. They conducted a protein-protein interaction network (PPI) of the DEGs, and they identified hub genes. To investigate the 44 hub genes’ prognostic values in CRC, they conducted a survival analysis using the sample splitting and Cox regression models based on the TCGA dataset. Their results showed that two down-regulated and two up-regulated hub genes were significantly associated with the CRC patients’ overall survival.

Bian *et al*. [7] analyzed data from four microarray datasets and identified DEGs from each of them. They identified the common genes across the four datasets, and this way, they obtained 53 genes. Then they utilized PPI, which identified ten essential genes according to their degree value, betweenness centrality, and closeness centrality. They used gene expression profiling interactive analysis (GEPIA) to apply survival analysis using the log-rank test based on the expression levels. Their results showed that four low expressed genes out of the ten genes were significantly related to unfavorable prognosis in the patients with CRC. Martinez-Romero *et al*. [14] identified a new set of gene markers associated with CRC to predict tumor progression and evolution towards inferior survival stages based on an integrated gene expression dataset of 1273 CRC samples. They compared the early and late stages of CRC using *limma* to identify the genes (2707 DEGs) that had a significant effect on CRC tumor progression. Then, they applied Kaplan-Meier to rank the genes based on the non-parametric log-rank test. Their results identified 429 essential genes in which overexpression is related to low survival rate and 336 crucial genes in which repression is associated with inferior survival. They validated the top 5 genes using an external cohort study and presented a good separation of the CRC samples into two low and high-risk groups.

A study by Pan *et al*. [13] proposed a predictive model based on RNASeq gene expression data. Their model uses the differentially expressed genes (DEGs) profiles. These profiles were obtained using the univariates and multivariate Cox regression, which was used to compare TNM stages to assess their predictive survival accuracy. Their results showed that 10 DEGs had a significant effect on CRC survival. Yan *et al*. [15] implemented random forests to identify biomarkers associated with survival in CRC based on a set of oligonucleotide microarray data. Their results showed that four genes had the potential to predict CRC survival.

To the best of our knowledge, RSF has not been used with gene expression data in the previous studies to predict CRC survival. The gene expression data is characterized by the problem of the curse of dimensionality and collinearity. To overcome this problem, the CRC survival is predicted based on selecting the differentially expressed genes (DEGs) in colorectal cancer that was based on the three-mutation status (KRAS, BRAF, and TP53) where they serve as a predictive biomarker of response to treatment in CRC. We assume that complex interaction between multiple DEGs contributes to prognostic survival differences between wild-type and mutant patients with CRC.

We developed and compared Cox proportional hazard (Cox PH) model and random survival forests (RSF) in predicting CRC survival and associated biomarkers using a public genome database from Gene Expression Omnibus (GEO). The aim was to assess the CRC survival predictors accounting for missing data based on the gene expression data. We selected 54 common differentially expressed genes from three mutations (KRAS, BRAF, and TP53), using the complete case samples, and performed analysis using Cox PH and RSF models before and after imputation.

## Materials and methods

### Dataset

**Fig 1:**
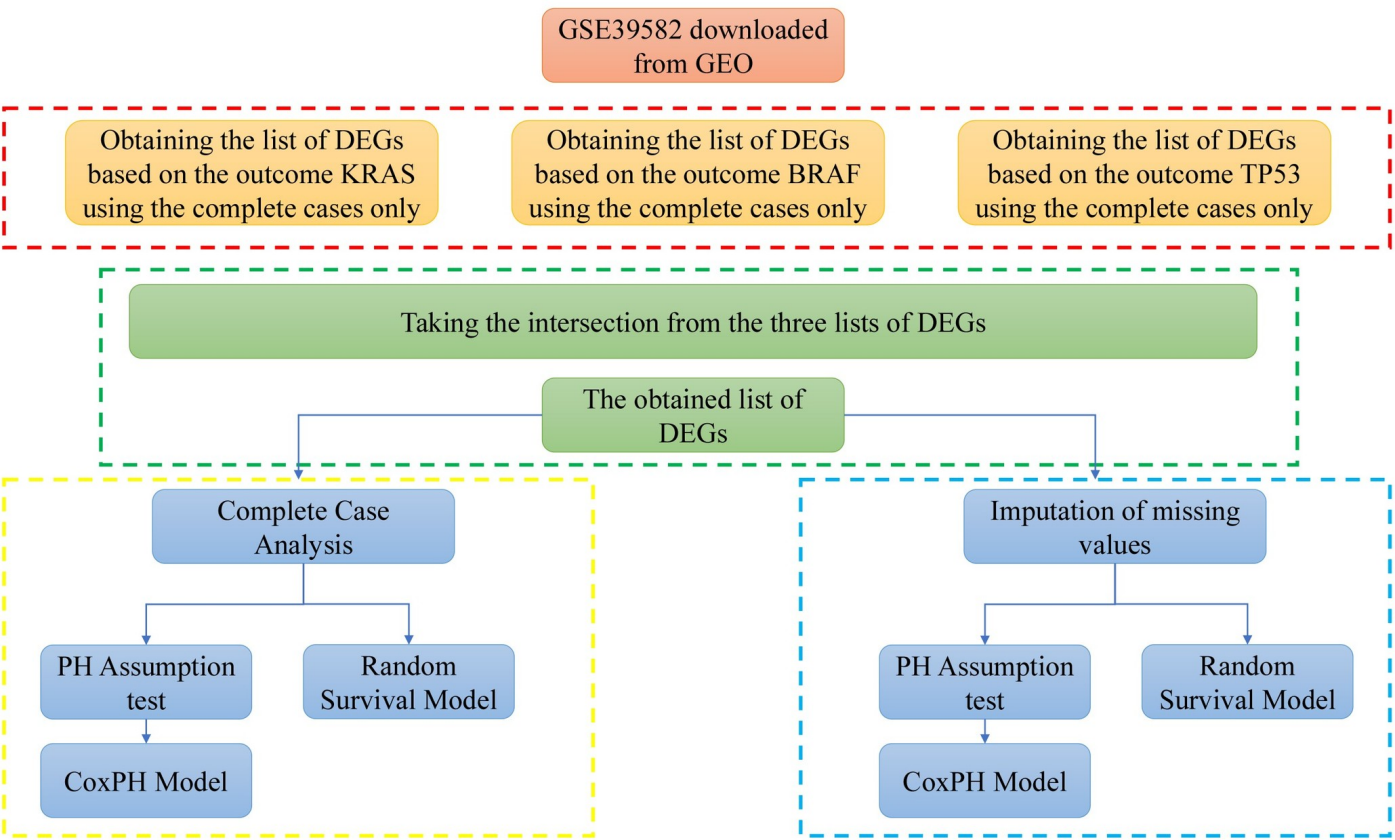
Flow-chart of the procedure followed in the pre-processing and analysis of the dataset.

The dataset with accession number GSE39582 [16], was downloaded from Gene Expression Omnibus (GEO) public database (https://www.ncbi.nlm.nih.gov/geo/) using the BRB-ArrayTools software (https://brb.nci.nih.gov/BRB-ArrayTools/). This dataset has 54675 probes taken from 566 samples with colon cancer and 19 non-tumor samples. Usually, the gene expression data includes noisy and or irrelevant genes. Therefore, performing data cleaning and feature (genes) selection are essential steps that should be applied before modeling the data. A pre-processing step was applied to prepare the dataset for modeling. These pre-processing steps are log2 transformation, quantile normalization, gene filtration, and differentially expressed genes analysis using a two samples t-test. Filtration is a process in data cleaning used to eliminate insufficiently expressed probes and those with excessive missing expression levels across the samples [17–20]. On the other hand, quantile normalization and log2-transformed steps to eliminate the variation between samples. BRB-ArrayTools is used to implement the filtration and normalization of the dataset. The two-sample t-test, with the 0.001 significance level threshold, was used for gene selection to provide informative genes for building survival models. The overall procedures that we followed in our analysis are summarized in Fig **1**.

### Statistical analysis

We analyzed the gene expression data using the *R* version (R-4.0.4). Summary statistics of the gene expressions are depicted in the supplementary file (see S1 Appendix). These statistics include the minimum, maximum, means, and standard deviations of the expression levels. We used frequency and percentages for the categorical data representing the clinical information, as shown in Table **1**. The statistical analysis was conducted in three phases; the first phase is the complete case analysis, followed by imputation of missing values in the outcome based on the covariates and an appropriate imputation model. Then we applied survival analysis on the complete case and imputed datasets. The survival analysis results on these two datasets were compared to evaluate the precision of estimates. Two separate models were fitted before and after imputations; the first is the Cox regression model, while the second is the random survival forests with log-rank and log-rank-score split rules. The missing values were assumed to be missing at random (MAR), where the probability of data being missing does not depend on the unobserved data, conditional on the observed data [21–24]; consequently, the genes and other covariates in the dataset were used to predict missingness.

**Table 1:** Clinical characteristics of colorectal cancer patients (N = 307).

Variable	Frequency (n)	Percentage (%)
**Age at diagnosis in years:** Mean (SD*)	66.8 (13.2)	
**KRAS Mutation**		
Mutant	123	40
WildType	184	60
**BRAF Mutation**		
Mutant	25	8
WildType	282	92
**TP53 Mutation**		
Mutant	166	54
WildType	141	46
**Tumor Location**		
Proximal	124	40
Distal	183	60
**Cancer stage**		
Early	156	51
Late	151	49
**Sex**		
Female	137	45
Male	170	55
**Molecular subtype**		
C1	65	21
C2	49	16
C3	43	14
C4	29	9
C5	29	9
C6	36	12

*SD: Standard deviation

#### Complete case analysis

The filtration step resulted in 18865 out of 54675 probes. These 18865 probes were used for further reduction analysis using a t-test. To find the differentially expressed genes (DEGs) that discriminate between the mutant and wild-type mutation, we used the three mutation types, KRAS, BRAF, and TP53. We created three different datasets using the 18865 probes with each of the three mutation types based on these three mutation types. First, we removed the samples with missing values for each of the three datasets according to their clinical outcome. Then, we calculated the correlation matrix for the gene expression data and filtered out one gene from every two genes that show a correlation coefficient greater than 0.6. Subsequently, we extracted three DEGs lists from all three datasets using a two-sample t-test based on 0.001 thresholds. Ultimately, from the three lists of DEGs, there were 54 common genes (see S1 Appendix). Also, we used the common samples across the three datasets to produce the complete cases in one dataset. The samples with missing or zero values in the event status and time variables were removed. We then converted the five TNM stages into a new categorical variable with two stages (Early and Late), where stages four and five were combined to give the late category. Finally, we used the obtained data for finding the most significant gene markers that may predict survival for CRC patients. **Table 2** provides a concise summary of the pre-processed data.

**Table 2:** Summary of the filtered datasets and the pre-processing steps.

Dataset (GSE39582) *		Number of samples	Complete cases	Common samples	Total number of genes	After filtration	Uncorrelated genes	DEGs (t-test)	Common genes
**Clinical outcomes**	KRAS	585	545	307	54675	18865	13827	711	54
	BRAF		512					2388	
	TP53		351					629	

* Three datasets with the same covariates and different clinical outcome

#### Multiple imputations of the missing values

**Fig 2:**
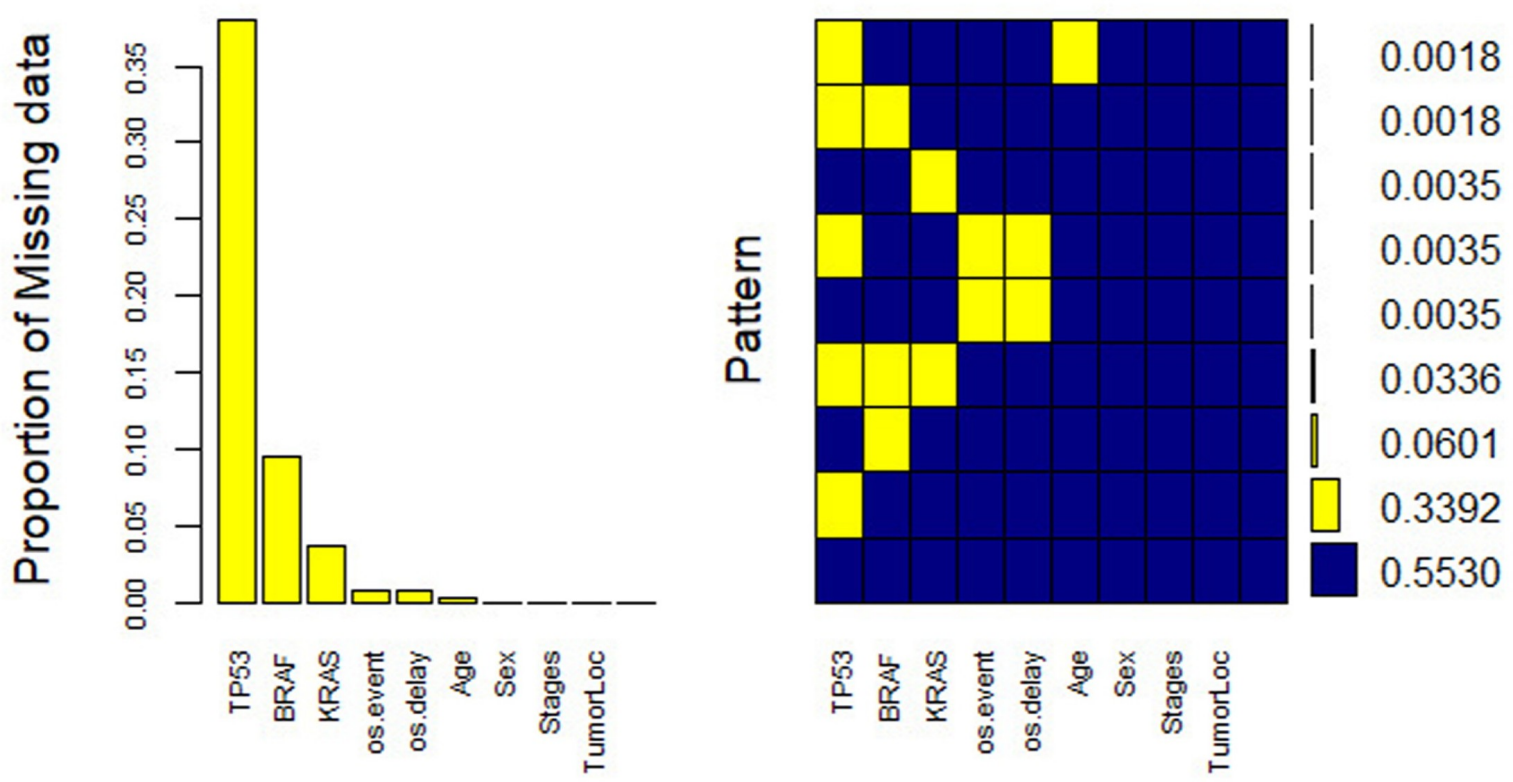
Proportion and patterns of missing values in the clinical characteristics available in the GSE39582 dataset.

To compensate for the missing data, we used the R package “mice (Multivariate Imputation by Chained Equations)”, which impute the missing values in the covariates. The mice package takes care of uncertainty related to missing values [23–25]. It assumes that the missing values are missing at random (MAR) see (Fig **2**), where the probability of missing data does not depend on the unobserved data, conditional on the observed data [21–24]. The mice package uses the genes and other covariates in the dataset to predict missingness. The missingness pattern in the data is assumed to be non-monotone. In this pattern, some subject values can be observed again after missing values happen [23–25]. For this missing data pattern, it is recommended to use the chained equations (fully conditional specification (FCS)) [26], or the Markov Chain Monte Carlo (MCMC) method to impute missing values [25].

We used FCS to handle the missing values in our dataset implemented in the mice package in *R* using a random forest model. The FCS is considered a powerful and statistically valid method for creating imputations in both categorical and continuous variables [26]. We generated 5 imputed datasets using random forest (rf) imputations after 100 iterations (imputation cycles). We used 1051991 as a random seed to replicate imputation results each time a multiple imputation analysis was performed. In addition, we followed the procedures indicated by the work of Sterne *et al*. [27] for reporting and analysis of missing data. KRAS, BRAF, TP53, and the event status were imputed as binary, while time and age imputed as numeric variables. The rest of the variables did not contain any missing values, and were used as auxiliary variables in the imputation model. Overall, firstly we performed a complete case analysis using Cox PH and random survival forests models. Thereafter, we compared the final models from this analysis to those from the multiply imputed dataset.

#### Experimental setup

To evaluate the different methods, the resulting dataset was divided into training set (80%) and testing set (20%). The training set was then divided into 10 subsets to train the methods using 10-fold cross validation approach to avoid overfitting. In the 10-fold cross-validation approach the integrated brier scores (IBS) is calculated on each fold left-out while the model is trained on the other 9 folds. Finally, the trained model is tested on the testing set. The model performance was measured using prediction error curve (pec).

### Statistical methods

#### Cox proportional hazard model (Cox PH)

Cox proportional hazard model is the most widely used statistical model for modeling time to event data [28]. The Cox PH evaluates the association of the survival time of patients and one or more predictors/genes variables. The Cox PH model relates the effect of predictors which include genes in our case to the rate or hazard of occurrence of an event such time to infection, death, recurrence of a condition at a certain point of time, this rate is generally referred as the hazard rate [29, 30]. In order to estimate the association of the gene expression levels and the survival time, consider *n* cancer samples say from sample *i* = 1,2,…,*n* and **g**_*i*_ = (*g*_*i*1_, *g*_*i*2_, *g*_*i*3_,…,*g*_*ip*_) is a vector of *p* genes expression level. The *i*^*th*^ patient survival data can be represented by (*T*_*i*_, *δ*_*i*_, *g*_*i*1_, *g*_*i*2_, *g*_*i*3_,…,*g*_*ip*_), where *i* = 1,2,…,*n*; *T*_*i*_ and *δ*_*i*_ indicate the survival time and the censor status respectively. The Cox PH model is mathematically represented as follow

$$h_{i}(t)=h_{0}(t)e^{\beta^{\prime }g_{i}}$$ (1)

where the parameters vector ***β***′ is the regression coefficients and ***g***_*i*_ is the covariates (genes) vector. The baseline hazard function *h*_0_(*t*) is unspecified and non-parametric function of an individual with all expression levels equal to zero [12, 31]. The model has a parametric part specified by the linear predictor and assumed to be proportional to the non-parametric baseline hazard. This means that for two individuals, *i* and *j*, the hazard ratio is

$$\frac{h_{i}(t)}{h_{j}(t)}=\frac{e^{\beta^{\prime }g_{i}}}{e^{\beta^{\prime }g_{j}}}$$ (2)

The hazard ratio is assumed to be independent of time *t*. The maximum partial likelihood method used to estimate the Cox PH model parameters is given by

$$L(\beta )=\prod_{r\in E}\frac{e^{\beta^{T}g_{r}}}{\sum_{j\in R_{r}}e^{\beta^{T}g_{j}}}$$ (3)

where E indicates the indices of the events (e.g., deaths) and *R*_*r*_ represents the vector of indices of the individuals at risk at time *t*_*r*_ - 0. The results of the Cox PH model are easy to interpret, however, there are key assumptions needed such as linearity and proportional hazards. We used *survival* and *survminer* packages to implement Cox PH model in *R*.

Moreover, we performed the stepwise regression for developing the Cox PH model at a 5% threshold level to find a simple model that shows the essential genes (markers) and clinical covariates correlated with the CRC. At each time, we removed the genes/ covariates that are not significant at *α* = 0.05 level of significance. Thereafter, we tested for the Cox PH assumption, and the integrative analysis of the CRC data showed five genes (markers) that passed the Cox PH assumption test. Thereafter, we used the five genes and the other clinical information to fit the Cox PH model.

#### Random survival forests (RSF)

Random survival forests are an ensemble of trees and a non-parametric method constructed by bagging of classification trees for right censored data [32, 33]. The RSF are an extension of the random forests method proposed by Breiman [34]. It works on high dimensional data where the number of covariates exceeds the number of the observations. Also it can handle data that consist of complex and non-linear relationships between the dependant and the independent variables and when the covariates violate the proportional hazard assumption [35]. There are several advantageous of using the RSF method, such as, it is not based on any model assumption compared to Cox PH model. It seeks to find a model that best represent the data in the case of limited survival data. In addition, it can handle high dimensional data unlike Cox PH, and it is robust to outliers in the explanatory variables [33]. RSF employs two steps of randomizations to grow the tree. These two steps are the bootstrap sample to select cases randomly and random selection of subset of covariates for splitting the nodes of the tree. These two steps help to decorrelate the tree [20, 33]. The RSF was implemented using the *randomForestSRC* package in *R* [36].

#### Random survival forests algorithm

We used the RSF algorithm that was introduced in the work of Ishwaran *et al*. [32] as shown below:

For *i* in 1: *ntrees*

- Draw bootstrap samples from the original total number of samples. For each bootstrap exclude approximately 37% of the samples as out-of-bag (OOB) samples.
- Build a survival tree for every bootstrap sample by recursively repeating the following steps for each node in a tree
  ○ Randomly select *v* genes at random from the *p* genes ($v=\sqrt{p}$)
  ○ To split the node, pick the best gene among the *v* genes, that maximizes survival differences between daughter nodes. We used log-rank and log-rank-score splitting rules as measures of survival differences.
  ○ Produce the tree to full size under the constraint that a terminal node should have no less than *d*_0_>0 unique deaths.
  ○ Calculate a cumulative hazard function (CHF) for every tree. Average the CHF for all the *ntrees* trees to find the ensemble CHF.
  ○ Calculate the OOB prediction error for the ensemble CHF, using OOB samples.

Once the survival tree is built, the ends of the tree are called the terminal nodes. Assume, the terminal node is *h* and *t*_*n*,*h*_ is the individual’s death time at node *h*, *d*_*n*,*h*_ is the number of deaths, and *M*_*n*,*h*_ is the number of individuals at risk at time *t*_*n*,*h*_. Therefore, the cumulative hazard function (CHF) can be estimated using the Nelson-Aalen estimator [37] as follows

$${\hat{H}}_{h}(t)=\sum_{t_{n,h}\leq t}\frac{d_{n,h}}{M_{n,h}}$$ (4)

The CHF was calculated for all the terminal nodes. The CHF for new observation *i* given a vector of genes as a covariate **g**_*i*_, can be calculated for one tree as follows

$${\hat{H}}_{h}(t|g_{i})={\hat{H}}_{h}(t),\;\mathrm{for}\;g_{i}\in h$$ (5)

To compute an ensemble CHF, the average of the *ntrees* trees is calculated, and the bootstrap ensemble CHF for an observation *i* is

$${\hat{H}}_{e}(t|g_{i})=\frac{1}{ntrees}\sum_{b=1}^{ntrees}{\hat{H}}_{b}(t|g_{i})$$ (6)

let,

$$I_{i,b}=\left\{ \begin{array}{l} 1\;if\;i\;is\;an\;OBB\;observation\;for\;ntrees\;training\;sample.\\ 0\;Otherwise. \end{array}\right.$$ (7)

then the OOB ensemble CHF for an observation *i* is given by

$${\hat{H}}_{e}^{*}(t|g_{i})=\frac{\sum_{b=1}^{ntrees}I_{i,b}{\hat{H}}_{b}^{*}(t|g_{i})}{\sum_{b=1}^{ntrees}I_{i,b}}$$ (8)

therefore, ${\hat{H}}_{e}^{*}(t|g_{i})$ is an average over the training samples where *i* is an OOB observation.

#### Log-rank split rule

The log-rank split-rule is a measure of a node separation which helps in determining the best split for that node [38]. Let *h* be a node of a tree and let there are *n* individuals with this node. Suppose (*T*_1_, *σ*_1_), (*T*_2_, *σ*_2_), …, (*T*_*n*_, *σ*_*n*_) are the survival outcomes corresponding to the n individuals. Thus, the best split at node *h* on covariate *x* at split point *c*, is the one that maximize the log-rank statistic between the two daughter nodes [32] given as follow

$$L(x,c)=\frac{\sum_{i=1}^{N}(d_{i1}-Y_{i1}\frac{d_{i}}{Y_{i}})}{\sqrt{\sum_{i=1}^{N}\frac{Y_{i1}}{Y_{i}}(1-\frac{Y_{i1}}{Y_{i}})(\frac{Y_{i}-d_{i}}{Y_{i}-1})d_{i}}}$$ (9)

The aim is to maximize the log-rank statistic by finding values of x and c that maximize *L*(*x*, *c*). Specifically, we are looking to find a predictor x* and c* such that |*L*(x*, c*)|≥|*L*(*x*, *c*)| for every x and c. This process is repeated at every node until the terminal node is reach.

#### Log-rank-score split rule

The log-rank-score split rule is a version of the log-rank-score split rule [39]. Consider *r* = (*r*_1_, *r*_2_,…,*r*_*n*_) as a vector that ranks the survival times (*T*, *δ*) = ((*T*_1_, *σ*_1_), (*T*_2_, *σ*_2_),…,(*T*_*n*_, *δ*_*n*_)) [39, 40]. Assume *a* = *a*(*T*, *δ*) = (*a*_1_(*r*), *a*_2_(*r*),…,*a*_*n*_(*r*)) indicates the ranked score vector. Let the ranked vector *r* order the genes variables in such a way that *g*_1_ < *g*_2_ < ⋯ < *g*_*n*_. Therefore, the log rank score for an observation at *T*_*i*_ is given by

$$a_{i}=a_{i}(T,\delta )=\delta_{i}-\sum_{j=1}^{\gamma_{i}(T)}\frac{\delta_{j}}{\left(n-\gamma_{j}(T)+1\right)},$$ (10)

where, $\gamma_{j}(T)=\sum_{i=1}^{n}\chi \{T_{i}\leq T_{j}\}$ is the number of individuals who died or were censored before or at time *T*_*j*_.

### Performance evaluation

We used integrated brier scores (IBS) measure [41] to assess and compare the accuracy of the predictive performance of all the models in this study. The IBS represent the average squared differences between the observed survival status and the predicted survival probability at time *t*. However, the value of the IBS is always between 0 and 1, the value of 0 represent the best possible IBS value. We calculated the brier scores (BS) measure using the test sample of size *n*_*test*_ as follows

$$BS(t)=\frac{1}{n_{test}}\sum_{i=1}^{n_{test}}\left\{{\left[0-\hat{S}(t|x)\right]}^{2}\frac{I(t_{i}\leq t,\delta_{i}=1)}{\hat{G}(t_{i}|x)}+{\left[1-\hat{S}(t|x)\right]}^{2}\frac{I(t_{i}>t)}{\hat{G}(t|x)}\right\}$$ (11)

where $\hat{G}(t|x)\approx P(C>t|X=x)$ is the Kaplan-Meier estimate for the conditional survival function of the censoring times. Therefore, the IBS is calculated as below

$$IBS=\int_{0}^{max(t)}BS(t)\;dt$$ (12)

## Results

### Cox proportional hazards analysis

The results of the survival problem based on gene expression data were obtained using *R*. We used the Cox PH model based on the selected covariates that satisfy the Cox PH assumptions. We tested the Cox PH assumptions using the Schoenfeld residual test implemented by the function *cox*.*zhp*. The Cox PH model assumes the regression parameters are constant over time. Therefore, the hazard ratios for any two individuals are constant over time. However, the covariates that do not satisfy the Cox PH assumptions do not meet the criteria to be entered in our final Cox PH model. As a first step, we fitted the Cox PH model for all the covariates (genes and clinical variables) in our dataset and then obtained the Cox PH assumption using the Schoenfeld residuals Table **3**. The genes and variables in violation of the Cox PH assumption (p<0.05) were DUSP4, SYTL1, and molecular subtype.

**Table 3:** Testing the proportional hazard assumption using scaled Schoenfeld residuals.

Probeset ID (Symbol)	χ^2^* (df)	p-value
204014_at (DUSP4)	10.219 (1)	0.0014
212947_at (SLC9A8)	1.345 (1)	0.2462
218611_at (IER5)	2.045 (1)	0.1527
219973_at (ARSJ)	3.601 (1)	0.0577
221522_at (ANKRD27)	1.583 (1)	0.2083
221605_s_at (PIPOX)	1.651 (1)	0.1988
227134_at (SYTL1)	4.699 (1)	0.0302
Age at diagnosis (years)	2.589 (1)	0.1076
Molecular subtype	15.824 (5)	0.0074
Disease stages	1.173 (1)	0.2787
Sex	0.378 (1)	0.5388
Tumor location	0.951 (1)	0.3294

*Chi-square statistic

From the Cox PH model in Table **3**, three variables violated the Cox PH assumption, and therefore, these genes and molecular subtype were not included in the final Cox PH model. We fitted the Cox PH model on the genes and variables that did not violate the Cox PH assumptions before and after imputation. The results from this analysis are shown in Table **4**. Results before imputation of missing values indicated that *218611_at (IER5)* (HR = 9.51, 95%CI 1.30, 69.58), 221522_at (*ANKRD27*) (HR = 34.89, 95%CI 1.91, 635.90), and late disease stage (HR = 1.97, 95%CI 1.33, 2.93) were associated with higher hazards of death. However, we note that two confidence intervals for *IER5* and *ANKRD27* are quite wide; therefore, they should be interpreted caution. For every year increase, the hazards of death increased by 1.03 (95%CI 1.01, 1.05). Significantly lower hazards were observed in *212947_at (SLC9A8)* (HR = 0.09, 95%CI 0.02, 0.49), *219973_at (ARSJ)* (HR = 0.23, 95%CI 0.09, 0.58), and 221605_s_at (*PIPOX*) (HR = 0.43, 95%CI 0.22, 0.85) differentially expressed genes.

**Table 4:** Multivariable Cox PH results for predictors of colorectal cancer survival among adults aged 24 years and above.

Probeset ID (Symbol) / Variables	Before imputation (N = 307)			After imputation (N = 566)		
	HR* (SE)	95%CI	P-value	HR* (SE)	95%CI	P-value
**212947_at (SLC9A8)**	0.09 (0.84)	(0.02, 0.49)	0.005**	0.30 (0.66)	(0.08, 1.07)	0.066
**218611_at (IER5)**	9.51 (1.02)	(1.30, 69.58)	0.027*	6.48 (0.79)	(1.37, 30.53)	0.019*
**219973_at (ARSJ)**	0.23 (0.48)	(0.09, 0.58)	0.002**	0.44 (0.36)	(0.22, 0.89)	0.024*
**221522_at (ANKRD27)**	34.89 (1.48)	(1.91, 635.90)	0.016*	2.49 (1.06)	(0.31, 19.95)	0.393
**221605_s_at (PIPOX)**	0.43 (0.34)	(0.22, 0.85)	0.014*	0.49 (0.27)	(0.28, 0.83)	0.009**
**Age diagnosis (years)**	1.03 (0.01)	(1.01, 1.05)	0.001***	1.03 (0.01)	(1.01, 1.04)	<0.000***
**Sex**						
Female	1.00			1.00		
Male	1.23 (0.20)	(0.84, 1.81)	0.281	1.40 (0.15)	(1.05, 1.88)	0.024
**Stages**						
Early	1.00			1.00		
Late	1.97 (0.20)	(1.33, 2.93)	0.001***	1.96 (0.15)	(1.47, 2.63)	<0.000***
**Tumor location**						
Proximal	1.00			1.00		
Distal	1.06 (0.21)	(0.71, 1.58)	0.783	0.86 (0.16)	(0.63, 1.18)	0.356

HR: Hazard ratio, SE: Standard error, adjusted for 212947_at, 218611_at, 219973_at, 221522_at, 221605_s_at, age at first diagnosis, sex, disease stage, and tumor location.

After imputation of missing values, the Cox PH model showed that sex was a significant predictor of males having higher death hazards (HR = 1.40, 95%CI 1.05, 1.88) than females. Also, the disease stage covariate was a significant predictor where those with late disease stage had higher death hazards (HR = 1.96, 95%CI 1.47, 2.63) than early cases. Moreover, the results illustrated that *219973_at (ARSJ)* (HR = 0.44, 95%CI 0.22, 0.89), 221605_s_at (*PIPOX*) (HR = 0.49, 95%CI 0.28, 0.83) were related with lower hazards of death. For every year increase, the hazards of death increased by 1.03 (95%CI 1.01, 1.04). Significantly higher hazards were detected with gene *218611_at (IER5)* (HR = 6.48, 95%CI 1.37, 30.53) gene.

### Random survival forests analysis

We fitted two random survival forests models, including survival trees built using log-rank and the log-rank-score split rules on the datasets before and after imputation. These two models were built using the 54 genes and the other clinical information as covariates. The characteristics of the two fitted models are summarized in Table **5** below.

**Table 5:** Random survival forests results before and after imputation using log-rank and log-rank-score split rules.

	Before imputation (N = 246)*		After imputation (N = 453)*	
	Log-rank	Log-rank-score	Log-rank	Log-rank-score
Number of deaths	88	88	157	157
Number of trees	5000	5000	5000	5000
Forest terminal node size	15	15	15	15
Average no. of terminal nodes	13.58	11.92	25.34	22.14
No. of variables tried at each split	8	8	8	8
Total no. of variables	62	62	62	62
Resampling used to grow trees	swor	swor	swor	swor
Resample size used to grow trees	155	155	286	286
Analysis	RSF	RSF	RSF	RSF
Family	surv	surv	surv	surv
Splitting rule	log-rank	log-rank-score	log-rank	log-rank-score
Number of random split points	10	10	10	10
Error rate	41.26%	49.05%	33.22%	43.01%

* Analysis performed using the 80% training set

**Fig 3:**
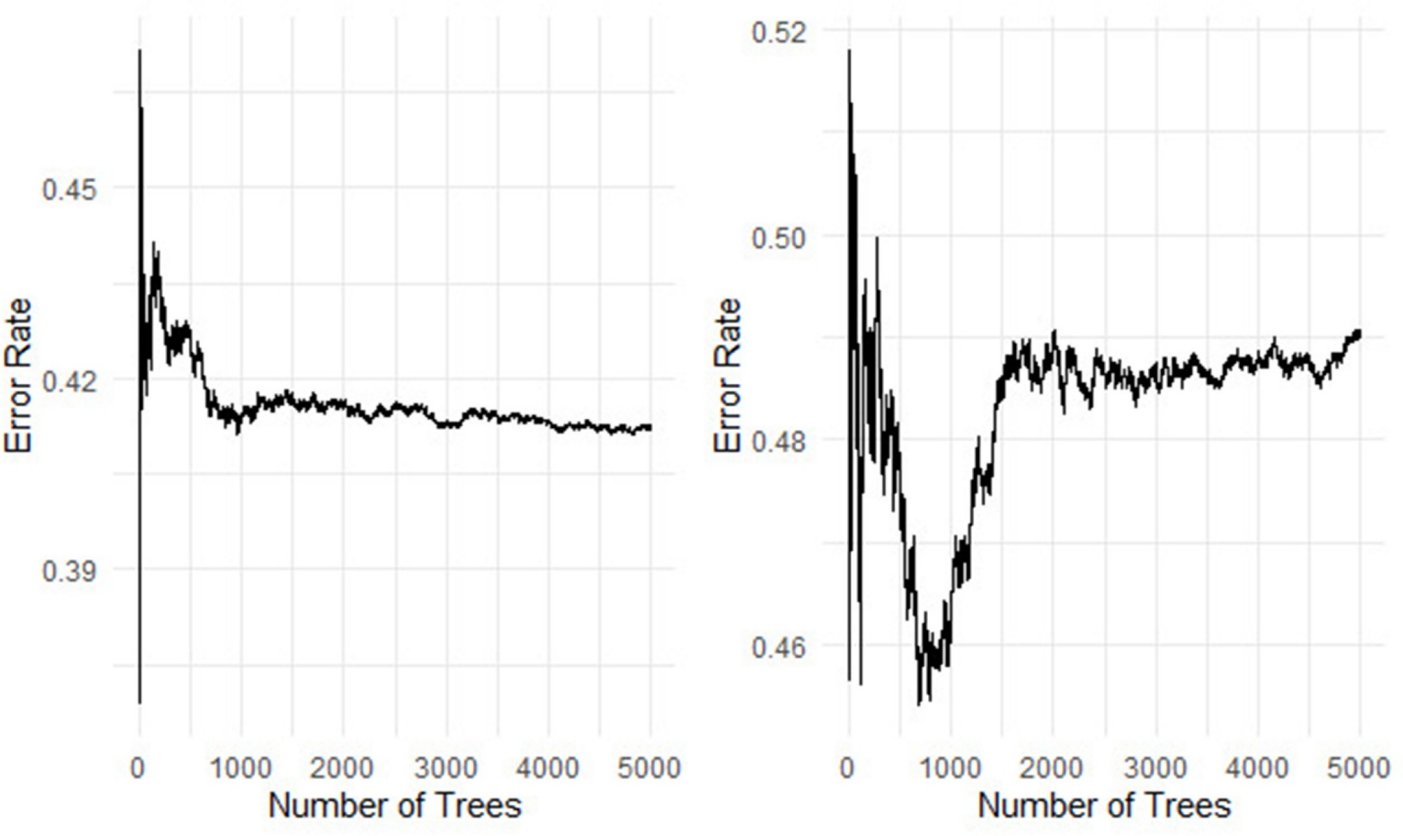
The prediction error rate for the random survival forests of 5000 trees before imputation and the log-rank and log-rank-score in the left and right panel used 80% training dataset.

**Fig 4:**
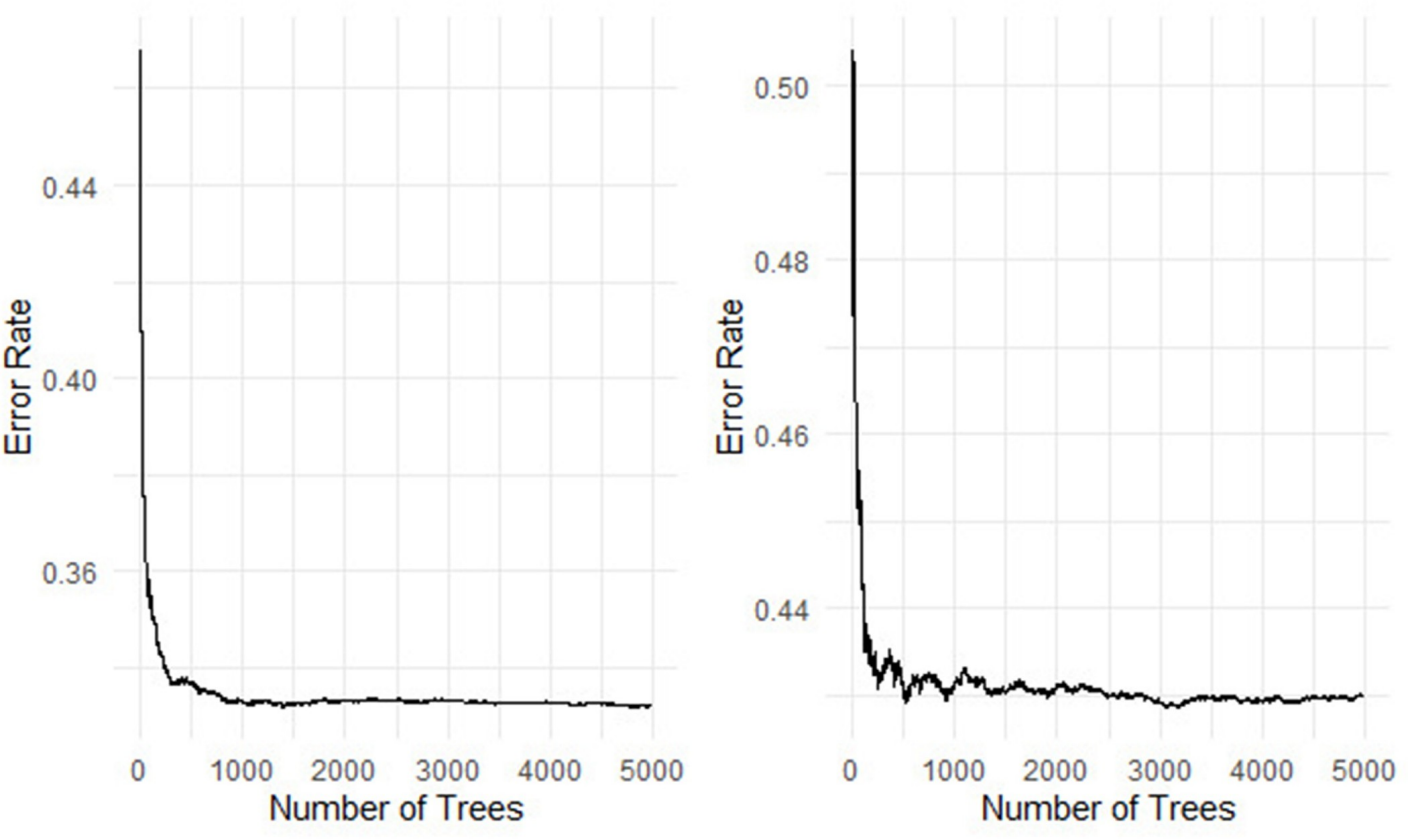
The prediction error rate for random survival forests of 5000 trees after imputation and the log-rank and log-rank-score in the left and right panel, respectively, using 80% training dataset.

Permutation importance measure used to identify the most important genes/ clinical variables associated with the survival of the colon patients [42–44]. We fitted a random survival forest model before imputation and after imputation with 5000 survival trees built using log-rank and log-rank-score and their results presented in Figs **3** and **4**.

Table **5** and Fig **3** show that the log-rank split-rule is more stable than the log-rank-score split-rule. Moreover, we fitted the model with 1000, 2000, and 3000 survival trees and noticed that the log-rank-score spilt-rule needs more survival trees to stabilize. In addition, the error rate for the forest built with survival trees based on the log-rank and log-rank-score split-rules are 41.26 and 49.05, respectively. These error rates of the RSF before imputation are much higher than the error rates for RSF built after imputation, as shown in Table **5**. This result indicates that the imputation can improve the performance of RSF.

**Fig 5:**
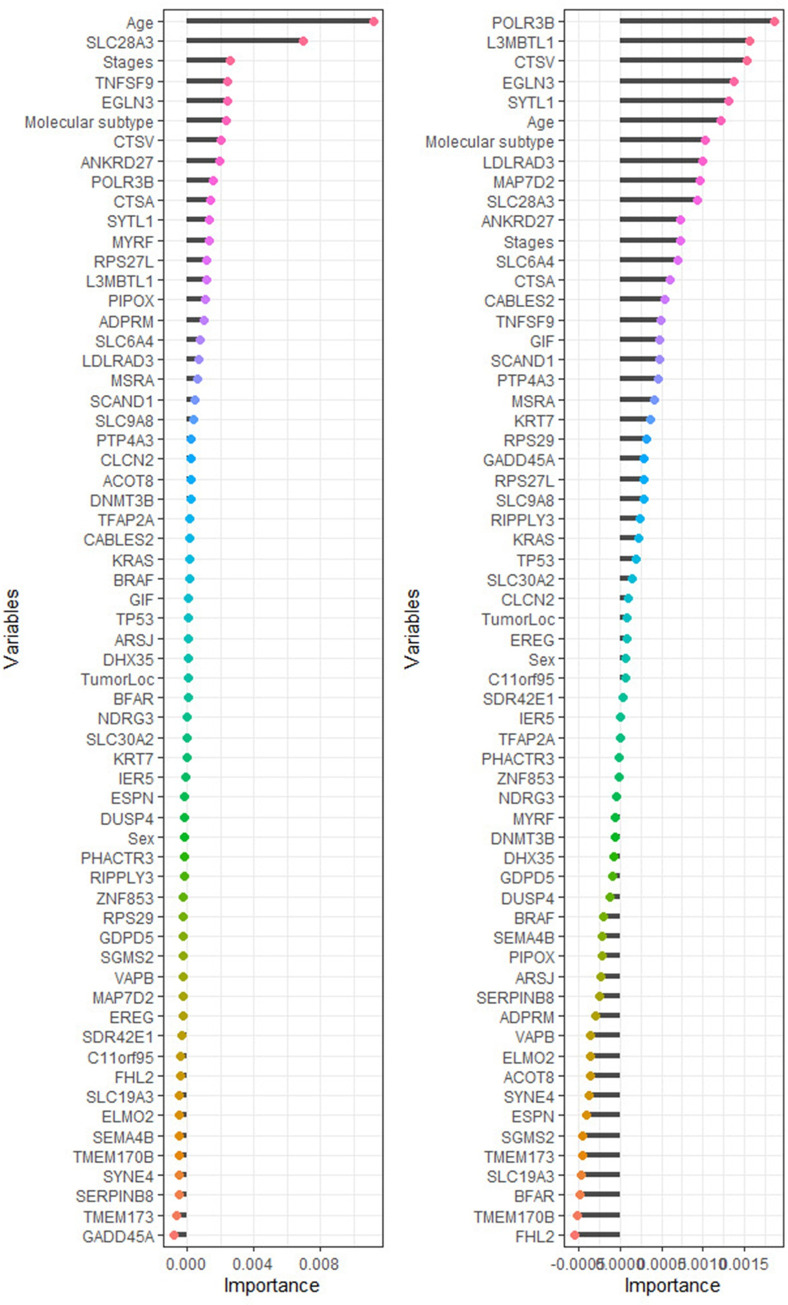
The rank of most predictive genes and clinical variables for colorectal cancer patients’ survival before the imputation is based on how they influence the survival outcome. The variables importance is built using log-rank and log-rank-score split-rules in the left and right panel, respectively.

**Fig 6:**
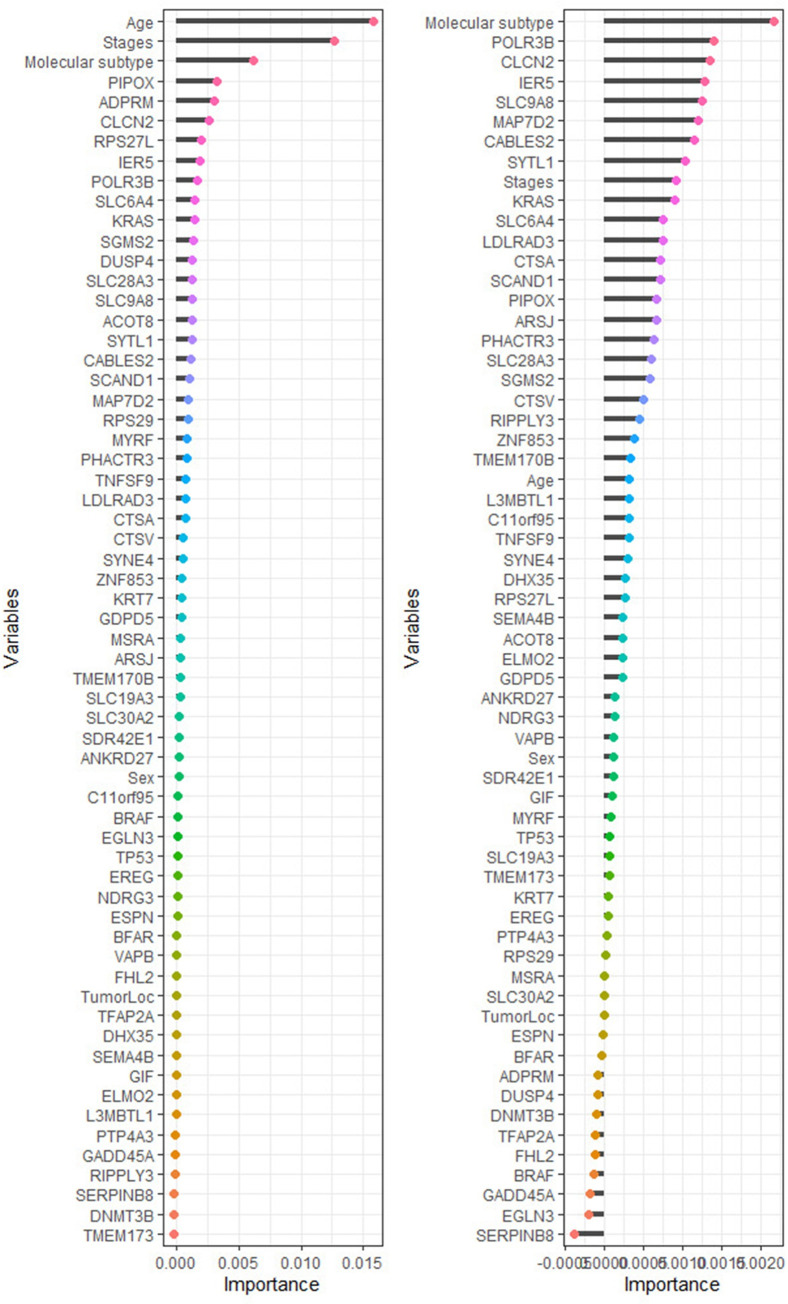
The rank of most predictive genes and clinical variables for colorectal cancer patients’ survival after the imputation is based on how they influence the survival outcome. The variables importance is built using log-rank and log-rank-score split-rules in the left and right panel, respectively.

The genes/ covariates associated with CRC ranked using RSF according to their importance before and after imputation based on the log-rank, and log-rank-score split-rules are presented in Figs **5** and **6**. Using RSF allows all 54 genes and other covariates regardless of their satisfying the Cox PH assumption. However, this is a very important characteristic of the RSF, as explained in the model building stage. The selection of the genes/ covariates in the model does not need to satisfy the too restrictive Cox PH assumption. RSF is purely non-parametric; hence there is no requirement of the Cox PH assumption being satisfied a prior.

We implemented RSF with 5000 survival trees built using two split-rules before and after imputation. The RSF identified the most important genes/ covariates that explain the survival of CRC patients by calculating the measure of the permutation importance as a variable’s importance [32, 43]. For the RSF before imputation see (Fig **5**), the top 20 genes/ covariates that are most important and strongly associated with the CRC obtained using the log-rank split-rule are age, *SLC28A3*, stages, *TNFSF9*, *EGLN3*, molecular subtype, *CTSV*, *ANKRD27*, *POLR3B*, *CTSA*, *SYTL1*, *MYRF*, *RPS27L*, *L3MBTL1*, *PIPOX*, *ADPRM*, *SLC6A4*, *LDLRAD3*, *MSRA*, and *SCAND1*. While the top 20 genes/ covariates that were identified by RSF using log-rank-score are *POLR3B*, *L3MBTL1*, *CTSV*, *EGLN3*, *SYTL1*, age, molecular subtype, *LDLRAD3*, *MAP7D2*, *SLC28A3*, *ANKRD27*, stages, *SLC6A4*, *CTSA*, *CABLES2*, *TNFSF9*, *GIF*, *SCAND1*, *PTP4A3*, and *MSRA*.

However, for the RSF after imputation (Fig **6**), the top 20 genes/ covariates strongly related to CRC identified using RSF with log-rank split-rule are age, stages, molecular subtype, *PIPOX*, *ADPRM*, *CLCN2*, *RPS27L*, *IER5*, *POLR3B*, *SLC6A4*, *KRAS*, *SGMS2*, *DUSP4*, *SLC28A3*, *SLC9A8*, *ACOT8*, *SYTL1*, *CABLES2*, *SCAND1*, and *MAP7D2*. Although the RSF with log-rank-score obtains a top 20 genes/ covariates strongly relevant to CRC, these genes/ covariates are molecular subtypes, *POLR3B*, *CLCN2*, *IER5*, *SLC9A8*, *MAP7D2*, *CABLES2*, *SYTL1*, stages, *KRAS*, *SLC6A4*, *LDLRAD3*, *CTSA*, *SCAND1*, *PIPOX*, *ARSJ*, *PHACTR3*, *SLC28A3*, *SGMS2*, and *CTSV*.

The RSF with log-rank split-rule after imputation performed better in terms of the error rate. Age and disease stage were the most important covariates that affecting CRC. However, the *PIPOX*, *IER5*, and *SLC9A8* were among the most important genes strongly associated with CRC. These results agree with the results achieved from fitting the Cox PH model presented in Table **4**. As far as significant effects are concerned, the most striking result to emerge was that the RSF model did pick other genes and covariates as substantial, e.g., molecular subtype and *DUSP4* which could not be included in the Cox PH model because of not satisfying the Cox PH assumption.

### Predictive performance

**Fig 7:**
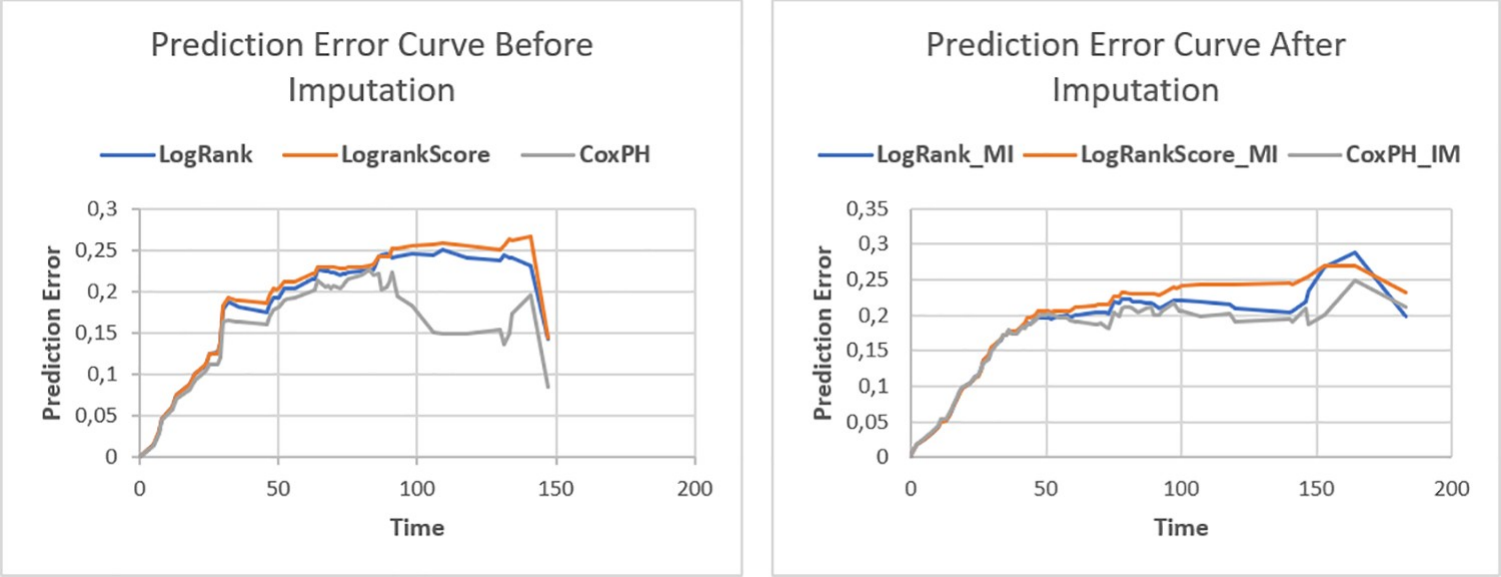
RSF with (log-rank and log-rank score) and Cox PH prediction error curve using 20% test set. The complete case and imputed dataset plots are in the left and right panel, respectively.

**Fig 8:**
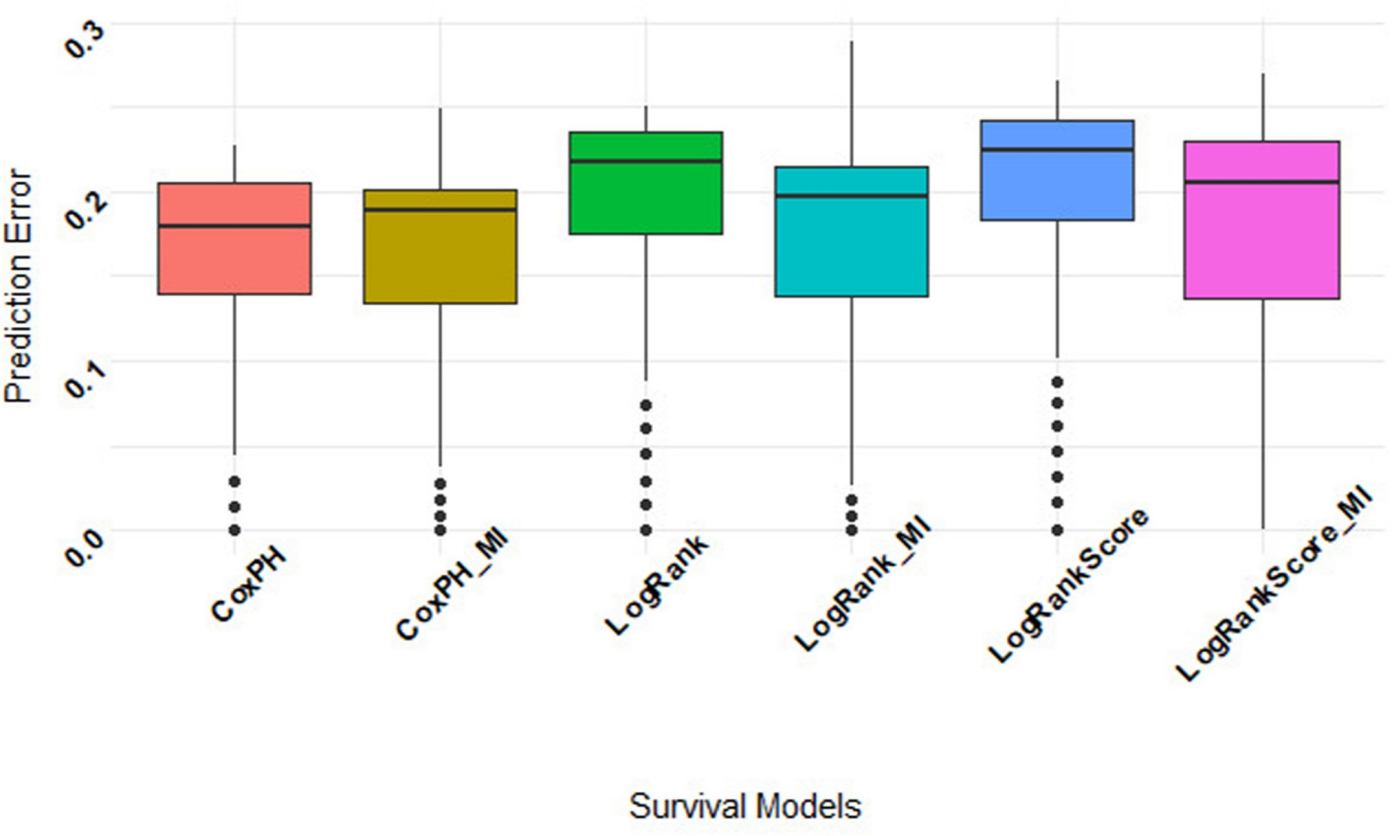
RSF with (log-rank and log-rank score) and Cox PH boxplot prediction error using 20% testing set together with the complete case dataset and the imputed data.

We assessed the predictive performance of the models using the integrated brier scores measure in *R* using the *pec* package [45, 46]. The model with lower prediction error rates is therefore considered useful [43, 47]. Figs **7** and **8** show the prediction error curve of the RSF (log-rank and log-rank score) and Cox PH models before and after imputation. These prediction curves show that Cox PH outperformed RSF with log-rank and log-rank score split rules. The Cox PH model before and after imputation had similar prediction errors, while RSF models under the two split-rules (log-rank and log-rank-score, respectively) after imputation had lower prediction error rates compared to before imputation as can be seen (Fig **8**). Their predictive performance exhibited that the log-rank split-rule is better than the log-rank-score split-rule. Moreover, we noticed that the Cox PH model showed good predictive performance compared to the two RSF under the two split-rules before and after imputation models. Thus it is safer to say that if all covariates satisfy the Cox PH assumption, the Cox PH model can be used [44].

Although the Cox PH model before and after imputation had better performance in terms of the prediction error rate, we can still not use it in the event of a violation of the proportionality of hazards assumption. Thus, in the presence of the non-proportional hazards genes/ covariates, using RSF is an appealing option in the analysis of survival data, especially for high dimensional genomics data. Genomics data are usually presented in a matrix, with the columns indicating the samples and the rows showing a genomic feature such as genes [48].

Table 6 shows a comparison of the model performance using the integrated brier scores. We can notice that the prediction error estimates are lower for RSF, especially in the case of using the log-rank as a split rule. In addition, RSF models perform substantially better than Kaplan-Meier and Cox PH models.

**Table 6:** Comparison of the models using the integrated brier scores.

Methods	Before Imputation	After Imputation
Kaplan Meier	0.199	0.201
RSF (Log-rank)	0.192	0.198
RSF (Log-rank score)	0.198	0.202
Cox PH	0.228	0.212

## Discussion

Cancer incidence and mortality are rapidly growing worldwide, exerting big physical, emotional, and financial problems on individual, families, communities, and health systems levels. Cancer is the first or second leading cause of death in 112 countries and is considered the third or fourth in 23 countries [3]. According to estimates from the World Health Organization (WHO), cancer is the leading cause of death around the world and accounting for nearly 10 million deaths in 2020. Moreover, WHO reported that CRC is the third common new cases, and it is also the second leading cause of death worldwide since 2020 [49]. The study aimed to determine the association between the genes and clinical covariates with CRC survival in the presence of missing values data. We also compared the predictive performance of the Cox PH and RSF models. The study provides essential information for CRC early detection and diagnosis.

The traditional regression-based methods to analyse survival data usually suffer from many problems such as restrictive assumptions including the proportionality, multicollinearity, curse of dimensionality, and lack of ability to rank the predictive performance. However, RSF models are frequently becoming a successful alternative for the analysis of the time to event data. In particular, the RSF is viewed as an appropriate analysing model for survival data, especially when the proportional hazards assumption is violated [39, 50]. When it comes to CRC survival analysis the gene expression and clinical information are utilized as covariates. The gene expression data contains many genes and most of these genes do not discriminate between normal cells and tumors. Therefore, we select the genes in which the change or difference in read counts between two conditions of experiment is statistically significant and such genes are known as the differentially expressed genes. In this study, the differentially expressed genes were obtained using three mutations based on the complete cases. The preliminary analysis showed that 54 potentially differentially expressed genes could be correlated with CRC survival and important for understanding the initiation and progression of CRC. The differentially expressed genes together with the clinical data were used to compare the predictive performance of the Cox PH model and RSF model before and after imputation on the CRC gene expression data.

We used stepwise regression for developing the Cox PH model at a 5% threshold level to get a simple model capturing the association between the top genes and CRC patient survival. Only five genes did not violate the Cox PH assumption in the final Cox PH model. The results show that the error rates of the RSF before imputation are much higher than the error rates for RSF built after imputation. Thus, the imputation can improve the performance of RSF. Although the Cox PH model had a better performance than RSF, the results from the current study demonstrate that the random survival forests models are more flexible than the models based on the Cox PH assumption as a prerequisite for variable inclusion in the model.

After imputation, the Cox PH model indicated *SLC9A8* and *ANKRD27* genes were no longer significant predictors of CRC survival. This because it is expected that the number of observations to increase, hence, statistical power to detect an effect. The variables that were not statistically significant before imputation may now be seen as statistically significant and vice versa. Therefore, this might affect the statistical power of some variables after imputation. Overall, the most prominent finding to emerge from the analysis based on Cox PH is that for one year increase in age, the hazards of death increase by 1.03, also the males are the most exposed to the hazards of death compared to females. Thus, this study supports evidence from previous observations [51–55].

The results of the RSF using both split-rules before and after imputation identified other genes/ covariates such as molecular subtype, *SLC6A4*, *KRAS*, *SGMS2*, *DUSP4*, and *SLC28A3*. These genes/ covariates show up as important in explaining CRC survival rates. However, these genes/ covariates did not appear very strongly associated with CRC survival in the Cox PH model. Thus, one interesting finding to note is that RSF models give additional information about variable importance.

Furthermore, the results from the two RSF models before and after imputation show that age, stages, molecular subtype, *SLC9A8*, *IER5*, *ARSJ*, *ANKRD27*, and *PIPOX* greatly affected the CRC mortality rates. These are ranked in the top 20 variables important in the two RSF models and agree with the Cox PH model results. Contrary to expectations, the RSF model did not pick sex as an important variable, while it is significant in the Cox PH model.

The Cox PH model had a better predictive performance in the presence of only those covariates that satisfy the Cox PH assumption compared to the RSF models. This result provides further support for the hypothesis that the Cox PH model works best under this assumption. In contrast, the out-of-bag error rate for the RSF with (log-rank and log-rank-score) before imputation is higher than that after imputation. This result implies that the imputation of missing values is a critical step and enormously improves the model’s performance.

The most striking result to emerge from the analysis of the RSF is that log-rank has a better performance compared to the log-rank-score split-rule [44]. However, with more survival trees the log-rank-score seems to be stabilize compared to a smaller number of survival trees.

We presented the development and validation of a robust five-gene signature (*SLC9A8*, *IER5*, *ARSJ*, *ANKRD27*, and *PIPOX*), which predicted overall survival (OS) for CRC patients. This gene signature was captured using Cox PH and RSF models based on two different scenarios. However, our study results successfully confirmed genes (markers) associated with CRC directly and identified new markers to enrich the field’s literature further. Furthermore, the results support previous studies such as Mohammed *et al*. [56], where age, sex, and stages were also shown to be related to CRC survival.

## Conclusion

Colorectal cancer (CRC) is a major cause of morbidity and mortality worldwide annually, making CRC the fourth common cause of death from cancer. However, the incidence of CRC has been steadily growing around the world, especially in developing countries. Therefore, the recent advances in technologies such as microarrays allowed for early detection screening using the individual’s gene expression profiles.

The present study was designed to identify the genes prognosis of CRC. We developed a robust gene marker associated with the CRC overall survival based on gene expression data generated from microarray, using Cox PH and RSF models before and after missing data imputation. The most prominent finding to emerge from this study is that the Cox PH model identified five genes (*SLC9A8*, *IER5*, *ARSJ*, *ANKRD27*, and *PIPOX*) related to CRC overall survival in addition to age, sex (after imputation), and clinical stages. The RSF model further confirmed these results and had five additional gene markers predicting CRC survival. In addition, imputation improved the model’s performance, and the current findings support the relevance of the missing data imputation. In summary, we recommend using a random survival forests model for survival data, especially in the high dimensional data where many genes might violate the Cox PH assumption.

## Supporting information

S1 Appendix Summary statistics of the 54 genes selected for survival analysis. (PDF)
