# Supplementary material for: Predictors of colorectal cancer survival using cox regression and random survival forests models based on gene expression data
Source: PLoS One. 2021 Dec 29;16(12):e0261625. doi: 10.1371/journal.pone.0261625 (PMC8716055; doi:10.1371/journal.pone.0261625)
Supplement: S1 Appendix — (PDF) [file pone.0261625.s001.pdf]

Predictors of colorectal cancer survival using Cox regression and random survival forests models based on gene expression data

Mohanad Mohammed[1,2*], Innocent B. Mboya[1,3], Henry Mwambi[1], Murtada K. Elbashir[4], Bernard Omolo[1,5,6]

[1]School of Mathematics, Statistics and Computer Science, University of KwaZulu-Natal, Pietermaritzburg, Private Bag X01, Scottsville 3209, South Africa.

[2]Faculty of Mathematical and Computer Sciences, University of Gezira, Wad Madani 11123, Sudan

[3]Department of Epidemiology and Biostatistics, Kilimanjaro Christian Medical University College (KCMUCo), P. O. Box 2240, Moshi-Tanzania.

[4]College of Computer and Information Sciences, Jouf University, Sakaka 72441, Saudi Arabia.

[5]Division of Mathematics & Computer Science, University of South Carolina-Upstate, 800 University Way, Spartanburg 29303, USA.

[6]School of Public Health, Faculty of Health Sciences, University of Witwatersrand, Johannesburg, South Africa.

* Corresponding Author, mohanadadam32@gmail.com

Table 1. Summary statistics of the 54 genes selected for survival analysis (N=307)

Probeset ID*	Gene Symbol	Min	Max	Mean (SD)
200661_at	CTSA	3.085296	3.537954	3.32 (0.09)
202949_s_at	FHL2	3.043399	3.51561	3.33 (0.08)
203725_at	GADD45A	2.841759	3.425452	3.10 (0.10)
204014_at	DUSP4	1.611363	3.343607	2.49 (0.36)
204073_s_at	MYRF	1.931487	3.118863	2.63 (0.25)
204653_at	TFAP2A	1.651537	3.271069	2.30 (0.34)
205767_at	EREG	1.330575	3.480803	2.71 (0.51)
206034_at	SERPINB8	2.2372	2.910791	2.57 (0.12)
206574_s_at	PTP4A3	2.234643	3.305859	2.77 (0.22)
206907_at	TNFSF9	1.82003	3.094666	2.34 (0.24)
207033_at	GIF	1.443817	3.342563	1.91 (0.30)
207267_s_at	RIPPLY3	1.415345	2.595129	1.84 (0.20)
207519_at	SLC6A4	1.860941	2.825926	2.20 (0.19)
209016_s_at	KRT7	1.894892	3.590677	2.42 (0.25)
210074_at	CTSV	2.406115	3.301904	2.87 (0.18)
210306_at	L3MBTL1	1.533169	2.845164	2.12 (0.25)
212947_at	SLC9A8	1.970226	2.87913	2.53 (0.13)
213499_at	CLCN2	2.205234	3.07652	2.58 (0.13)
218056_at	BFAR	2.900836	3.240013	3.08 (0.06)
218611_at	IER5	2.846535	3.438737	3.10 (0.11)
218641_at	C11orf95	2.273865	3.221723	2.87 (0.16)
219232_s_at	EGLN3	1.995892	3.25081	2.63 (0.23)
219281_at	MSRA	2.463159	3.096048	2.85 (0.10)
219459_at	POLR3B	2.164917	3.010461	2.75 (0.11)
219973_at	ARSJ	1.477639	2.882906	2.17 (0.25)
220363_s_at	ELMO2	2.207412	2.797861	2.52 (0.11)

220606_s_at	ADPRM	2.198647	2.884304	2.53 (0.13)
220668_s_at	DNMT3B	2.078965	2.929752	2.52 (0.15)
220736_at	SLC19A3	1.541837	3.07391	2.13 (0.32)
221522_at	ANKRD27	2.839777	3.303317	3.08 (0.08)
221605_s_at	PIPOX	1.599295	3.263056	2.21 (0.32)
224368_s_at	NDRG3	2.274761	3.136462	2.80 (0.16)
224916_at	TMEM173	2.209758	3.181648	2.79 (0.15)
225923_at	VAPB	2.391236	3.181648	2.80 (0.15)
226004_at	CABLES2	2.359722	3.15781	2.80 (0.13)
227134_at	SYTL1	1.970423	3.184009	2.67 (0.22)
227949_at	PHACTR3	1.159216	2.99362	1.86 (0.37)
228262_at	MAP7D2	1.340846	3.273704	2.27 (0.50)
229522_at	SDR42E1	2.066187	2.872696	2.53 (0.16)
230084_at	SLC30A2	1.996567	2.894033	2.32 (0.16)
232277_at	SLC28A3	1.367166	2.942343	2.07 (0.39)
232652_x_at	SCAND1	2.865234	3.359162	3.09 (0.10)
232884_s_at	ZNF853	1.813342	2.785865	2.15 (0.18)
233979_s_at	ESPN	1.901582	2.90773	2.39 (0.20)
234725_s_at	SEMA4B	2.722612	3.327446	3.08 (0.11)
234728_s_at	DHX35	1.992349	2.876806	2.47 (0.16)
234985_at	LDLRAD3	1.974432	3.167782	2.74 (0.21)
235515_at	SYNE4	1.868786	3.038119	2.57 (0.28)
235798_at	TMEM170B	1.180868	2.532567	1.82 (0.27)
236514_at	ACOT8	1.741381	3.000081	2.36 (0.25)
238824_at	RPS29	1.95044	2.952403	2.44 (0.14)
238935_at	RPS27L	1.804659	3.16445	2.60 (0.19)
242963_at	SGMS2	1.550172	2.834302	2.23 (0.22)
32502_at	GDPD5	2.424871	3.357056	2.86 (0.15)

*These genes were selected based on the intersection of the three outcomes (KRAS, PRAF, and TP53) using the T-test statistics from the original GSE39582 colorectal cancer data downloaded from the GEO repository.
